# Novel Bluetooth-Enabled Tubeless Insulin Pump: Innovating Pump Therapy for Patients in the Digital Age

**DOI:** 10.1177/1932296818798836

**Published:** 2018-09-21

**Authors:** Trang T. Ly, Jennifer E. Layne, Lauren M. Huyett, David Nazzaro, Jason B. O’Connor

**Affiliations:** 1 Insulet Corporation, Billerica, MA, USA

**Keywords:** diabetes, insulin pump, patch pump, tubeless pump, Omnipod

## Abstract

The Omnipod DASH™ Insulin Management System (Insulet Corp, Billerica, MA) is a discreet, tubeless, wearable insulin pump that holds up to 200 units of U-100 insulin and delivers therapy through customizable basal rates and bolus amounts. This recently FDA-cleared system consists of the insulin pump (“Pod”), which is worn on body and delivers insulin, and the Personal Diabetes Manager (PDM), which is a handheld device used to wirelessly control and monitor the Pod functionality. The PDM can also be paired with the CONTOUR^®^ NEXT ONE blood glucose (BG) meter (Ascensia Diabetes Care, Basel, Switzerland) to wirelessly receive BG readings. This review provides a detailed description of the Pod and PDM. Key features of the Pod are described, including the novel pump delivery mechanism, waterproof (IP28) housing design, and automated cannula insertion. The technology introduced in the new system, such as touchscreen PDM interface, Bluetooth^®^ wireless technology, and wireless internet connectivity, is also presented. Last, Omnipod^®^ Insulin Management System clinical data are reviewed, including early feasibility results for the Omnipod Horizon™ Automated Glucose Control hybrid closed-loop system.

In the spring of 2000, two colleagues, John Brooks III and Duane Mason, were seated next to each other on a flight headed home to Boston. John’s son Rob was diagnosed with type 1 diabetes at age 3 and wore a traditional tubed insulin pump. John lamented how frustrating and restrictive it was for his son to wear a pump with several feet of tubing. On the back of a napkin, John and Duane drew models of a disposable, cost-effective, mechanical insulin delivery device about the size of matchbox that could be discreetly worn directly on the body by people with diabetes. Within days, a patent was filed. Omnipod was born with $250 000 of seed investment from these two families, to make life better for Rob and other children with diabetes. The rest as they say, is history.

Today, their invention is the world’s first commercially available tubeless insulin pump, used by over 150 000 patients worldwide. Currently two systems are available including the Omnipod^®^ Insulin Management System in Europe, Canada, and the United States, as well as the recently FDA-cleared Omnipod DASH™ System (US only) as shown in Figure 1 (Insulet Corp, Billerica, MA). These systems stay true to John and Duane’s vision of discreet and precise insulin delivery. Since its first introduction to the market in 2005, the pump or “Pod” has been further miniaturized to be smaller and lighter than the original device whilst still maintaining the same 2 ml or 200 unit insulin reservoir. The components of the system have always included a disposable infusion pump (Pod) and the wireless remote controller device or Personal Diabetes Manager (PDM).^1,2^ The newly FDA-cleared system builds upon these innovations utilizing mobile technologies and Bluetooth^®^ wireless technology to enable digitalization of the PDM.

**Figure 1.**
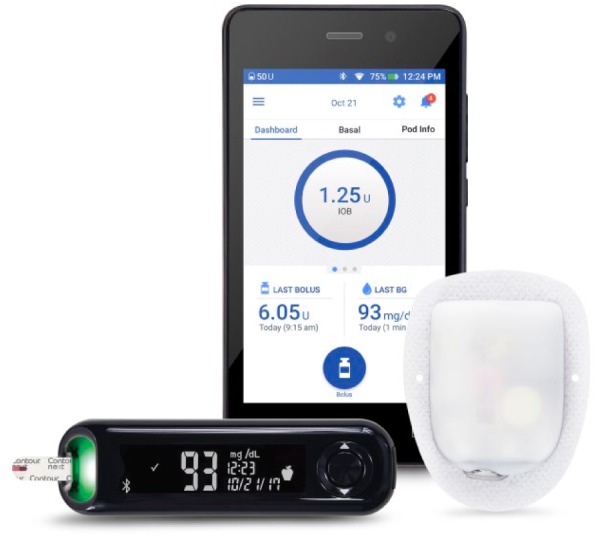
Omnipod DASH™ Insulin Management System Personal Diabetes Manager (PDM) and Pod, with the interoperable CONTOUR^®^ NEXT ONE blood glucose meter.

## Omnipod DASH Insulin Management System: The Pod

The newly FDA-cleared insulin management system (Figure 1) features the Pod, a lightweight, waterproof (IP28), self-enclosed insulin pump that is worn directly on the body for up to 72 hours. The Pod allows for continuous subcutaneous insulin infusion (CSII) via a soft in-dwelling cannula extending directly beneath the pump. A key differentiating feature of the Pod is the automated priming and cannula insertion, with the needle being neither handled nor seen by the user. The pump reservoir holds up to 200 units of insulin, and delivers insulin in increments of 0.05 unit. The Pod is a single-use disposable device that attaches to the body with a standard medical grade adhesive.

The Pod is intended for use with rapid-acting U-100 insulin: NovoLog^®^ (Novo Nordisk A/S, Copenhagen, Denmark), Humalog^®^ (Eli Lilly, Indianapolis, IA, USA), or Apidra^®^ (Sanofi-Aventis, Paris, France).

Although it is true that all insulin pumps are able to perform CSII, these devices should not be viewed as interchangeable. The newly FDA-cleared insulin management system described here is differentiated from other insulin pumps, with several features that were incorporated to improve the user experience and optimize patient engagement.

### Automatic Priming and Cannula Insertion

To activate the Pod, the user fills the Pod with a minimum of 85 units and maximum of 200 units of insulin, pairs the Pod with the PDM, removes the adhesive backing and places the Pod on the body. Using the PDM, the Pod is then activated with automated priming and cannula insertion. The cannula is inserted in 0.005 seconds. The steel insertion needle safely retracts inside the Pod and remains inaccessible to the user. A consistent insertion distance (6-7 mm) and angle (50 degree normal axis to skin) is achieved. Once the cannula is inserted, insulin delivery commences automatically through preprogrammed basal rates. Insulin delivery instructions are delivered securely through wireless, Bluetooth technology from the PDM to the Pod. Once the instructions have been communicated, the Pod will continue insulin infusion according to the preprogrammed rates, even if the PDM is out of range.

### Pod Structure and Design

The Pod is waterproof at a depth of 25 feet for up to 60 minutes (IP28). This allows users to bathe and swim without having to be disconnected from insulin therapy. The housing includes a viewing window at the infusion site, which allows the user to inspect the insertion site for proper cannula placement. A colored visual indicator on the top of the device also confirms successful cannula deployment. The Pod includes an air vent to ensure pressure equilibration with the surrounding environment, relevant for altitude and air travel. A piezo electric crystal generates audible alerts for reminders and hazard alarms.

### Pod Actuation Mechanism

**Figure 2.**
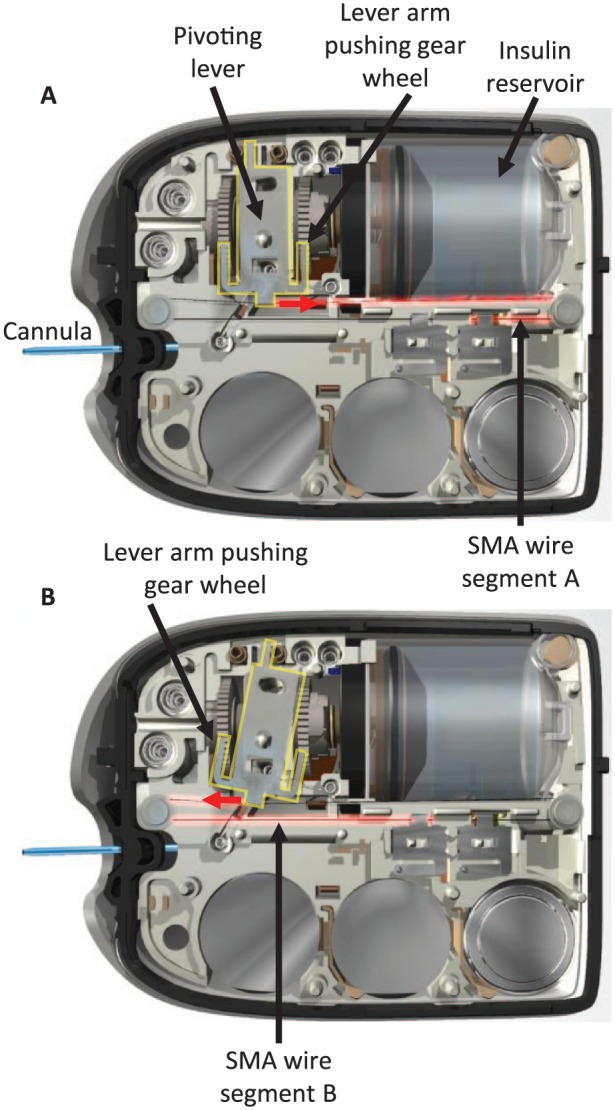
Schematic diagram showing the actuation mechanism of the Pod. Shape memory alloy (SMA) wire technology pulls the lever back and forth to deliver 0.05 units of insulin with each independent movement. Two wire segments are alternately heated (represented by red highlight on the wire, labeled A and B) to pivot the lever (indicated by yellow highlight). In diagram A, wire segment A is heated and contracts, causing the lever to pivot (indicated by red arrow) and push the gear wheel forward with the right arm (changing linear motion to rotational motion). In diagram B, wire segment A cools and elongates while wire segment B is heated and contracts, causing the lever to pivot in the other direction (indicated by red arrow) and push the gear wheel forward with the left arm.

The actuation mechanism of the Pod incorporates microprocessor control and a novel nitinol shape memory alloy (SMA) wire assembly with multiple redundant safety sensors (Figure 2). Stored electrical energy from a battery source is converted into mechanical motion in the SMA wire assembly. As an electrical current is applied to the wire, high resistance in the wire causes it to heat up, go through a phase change and contract due to the properties of nitinol. The heating and cooling process allows the wire to contract and expand. The linear motion from the SMA wire assembly is converted into rotational motion in a gear assembly, which then translates a leadscrew that is attached to a plunger/piston within the reservoir. The Pod is able to sense both linear and rotational movements. The translation of the plunger is what positively meters insulin from the reservoir, through the cannula and to the user. Each actuation of the SMA wire delivers a minimum dose increment of 0.05 units of insulin.

Advantages of this system include considerable cost reductions compared to direct current (DC) or solenoid motors, flexibility in design enabling a more compact insulin delivery system, weight reduction, and the inability of the SMA wire-actuated motor to run away when power is directly applied to the SMA wire assembly. The inherent design of the SMA wire actuated drive system makes the likelihood of runaway insulin delivery virtually impossible, as the maximum unintended dose would be one pulse, or 0.05 units.

## Personal Diabetes Manager

The PDM is a handheld, touchscreen, locked-down Android device that is used to remotely control insulin delivery and periodically monitor Pod status. The PDM device establishes secure, bidirectional, wireless communication with both the Pod and the CONTOUR^®^ NEXT ONE blood glucose (BG) meter (Ascensia Diabetes Care, Basel, Switzerland). The PDM screen measures 4 inches diagonal, and the device uses a rechargeable lithium ion battery. The PDM provides audible and vibratory alarms, alerts, and reminders related to insulin delivery, reservoir level, Pod functioning, and battery life.

### Secure Connectivity Between System Components

**Figure3.**
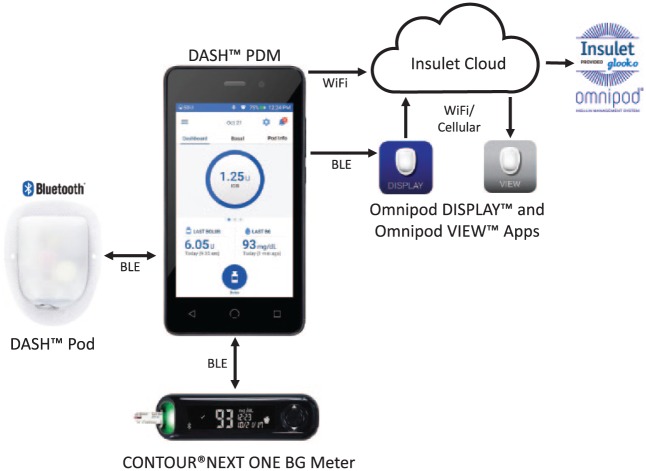
Omnipod DASH™ Personal Diabetes Manager (PDM), Pod, and integrated data communication systems. The PDM communicates with the Pod and the CONTOUR^®^ NEXT ONE blood glucose meter through Bluetooth^®^ wireless technology. The PDM uploads data to the secure Insulet Cloud via Wi-Fi, which can then be viewed on a personal cell phone using the Omnipod VIEW™ mobile application. The PDM can also communicate through Bluetooth wireless technology to the Omnipod DISPLAY™ mobile application installed on a personal cell phone. The Omnipod DISPLAY mobile application can then automatically upload data to the Insulet Cloud using Wi-Fi or cellular data. Data uploaded to the Insulet Cloud will automatically merge with the Glooko^®^ data management system to allow integrated data management.

The insulin management system employs a set of security procedures that mitigate various data communications and access risks to the system. Data encryption and authentication occurs with each Pod activation. Communication between the PDM and other system components, as well as the Insulet Cloud and other applications, is governed by these security procedures (Figure 3). Once activated, the Pod only responds to the paired PDM.

### Insulin Delivery

Insulin can be delivered continuously with a basal rate set per hour or as a bolus, which is delivered either immediately (Immediate Bolus) or over an extended period of time (Extended Bolus). Up to 12 basal programs can be set (presets) and each program may contain from one to 24 basal rates. The range of basal rates is from 0 to 30 units per hour. The user may also set temporary basal rates (Temp Basal) for a predefined duration from 30 minutes to 12 hours. The maximum bolus can be set from 0.05 to 30 units. Insulin is delivered by increments of 0.05 units at a maximum rate of 1.5 units per minute. After the insulin delivery instructions have been transmitted from PDM to Pod, the Pod will continue insulin infusion according to the preprogrammed rates, even if the PDM is out of range.

### Bolus Calculator Settings

The programmable settings for the bolus calculator include the insulin to carbohydrate ratio (IC ratio), correction factor, target glucose and duration of insulin action. The IC ratio defines how many carbohydrates are covered by one unit of insulin (range 1 to 150 g). The correction factor defines how much one unit of insulin lowers the blood glucose level and can be set between 1 to 400 mg/dL. Target glucose for bolus calculations may be set between 70 to 200 mg/dL. The duration of insulin action may be set between 2 to 6 hours. These settings allow a suggested amount of insulin to be delivered and these values should be programmed in consultation with the user’s health care provider.

### Insulin on Board

Insulin on board (IOB) is an estimate of the amount of insulin still active in the body from previous boluses. The calculation is dependent upon the duration of insulin action setting. The degradation of IOB follows a linear pattern. For instance, if the duration of insulin action is set at 4 hours and the previous bolus of 10 units was delivered 2 hours ago, the current IOB will be 5 units. This value will be subtracted from any suggested correction bolus at the current time to reduce the likelihood of insulin stacking.

### Blood Glucose Meter Interoperability

The newly FDA-cleared insulin management system is compatible with the CONTOUR NEXT ONE BG meter (Figures 1 and 3). Interoperability with the PDM is enabled through Bluetooth wireless technology. The user may accept the BG value from the meter through the wireless synchronization and then add this value to the bolus calculator. A BG value taken within the last 10 minutes will automatically be added to the bolus calculator.

### Food Library

The embedded Food Library allows users to search the nutritional content of a myriad of foods from an extensive database of over 80 000 branded and unbranded products. Users may add food items from the library directly to the bolus calculator where the estimated carbohydrate content is added to the bolus calculator. These tools are designed to encourage prandial bolus delivery for patients. Nutritional information such as carbohydrates, fiber, fat, protein, and sodium are easily accessible for users.

### Insulet Cloud

The newly FDA-cleared insulin management system has Wi-Fi-enabled connection to the Insulet Cloud, strictly egress in nature, to allow PDM data to be uploaded wirelessly (Figure 3). This functionality will be implemented during the full commercial launch. Access to these data will allow more comprehensive and efficient support for users and the health care providers. These data provide valuable insights to achieve better outcomes for users. PDM Wi-Fi connectivity is optional and is not required for the core function of insulin delivery.

### Mobile Applications

The PDM may also be connected through Bluetooth wireless technology to the Omnipod DISPLAY™ mobile application, downloadable to the user’s personal phone, which allows the user to view PDM information and automatically upload the data from the personal phone to the Insulet Cloud. PDM data that is uploaded to the Insulet Cloud can be utilized through remote applications including the Omnipod VIEW™ mobile application to allow caregivers to access information such as recent insulin delivery. The mobile applications allow uploaded PDM data to be viewed simultaneously with continuous glucose monitoring (CGM) sensor data from a Dexcom CGM (Dexcom Inc, San Diego, CA) on the user’s personal phone.

### Data Management

Data from the PDM that is automatically uploaded to Insulet Cloud will merge with the Glooko^®^ data management system (Glooko Inc, Mountain View, CA) to allow seamless integrated data management around full commercial launch. These data can be remotely reviewed by clinicians, caregivers, and patients for therapy adjustment and to optimize diabetes treatment. Similar to the previous Omnipod System, the Omnipod DASH System data can also be uploaded to Glooko at the provider’s office.

## Clinical Data

### How Does Tubeless Insulin Pump Therapy Compare to Other Treatment Modalities?

#### Type 1 Diabetes

**Figure 4.**
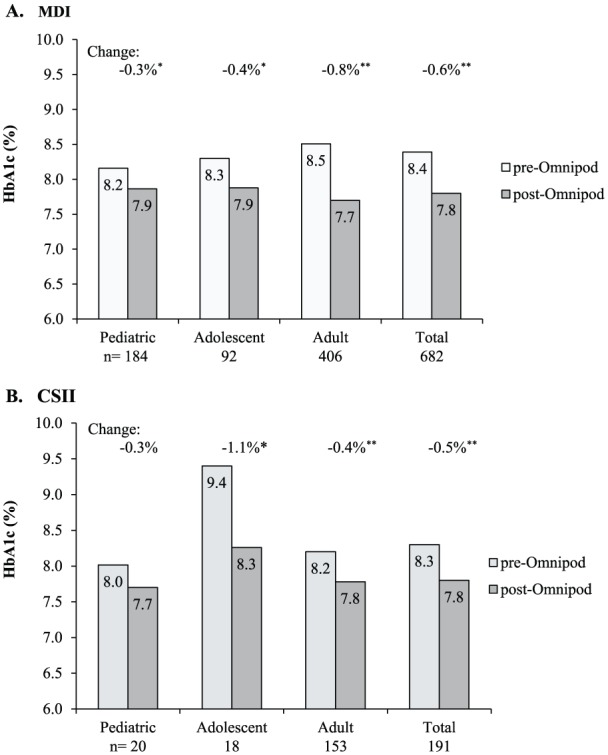
Change in HbA1c at 3 months post–Omnipod treatment initiation in patients with type 1 diabetes. (A) Change from previous treatment with multiple daily injections (MDI). HbA1c change (mean ± SD) was –0.3% ± 1.3, –0.4% ± 1.4, –0.8% ± 1.3, and –0.6% ± 1.3 for pediatric, adolescent, adult, and total, respectively. *P = .002. **P **<** .001. (B) Change from previous treatment with continuous subcutaneous insulin infusion (CSII). HbA1c change (mean ± SD) was –0.3% ± 0.8, –1.1% ± 1.6, –0.4% ± 1.1, and –0.5% ± 1.1 for pediatric, adolescent, adult, and total, respectively. *P **<** .01. **P **<** .001. Reprinted with permission from Layne et al.^3^

In a multisite, retrospective study of 873 pediatric, adolescent, and adult patients with type 1 diabetes in the United States, Layne et al.^3^ reported clinically meaningful improvement in glycemic outcomes for patients initiating therapy with the tubeless insulin management system compared to previous treatment with multiple daily injections (MDI) and tubed pumps. After 3 months of tubeless insulin pump use, an overall reduction in HbA1c of 0.6% was seen compared to baseline. For MDI patients, HbA1c improved from 8.4% to 7.8%. For previous tubed pump users, HbA1c improved from 8.3% to 7.8% (Figure 4). These improvements were seen across all age groups independent of prior treatment.

Another important finding from this study was the reduction in the total daily dose (TDD) of insulin with tubeless insulin pump use reported for both previous MDI and tubed pump users. There was a 16.4% decrease in TDD of insulin at 3 months for the total population. This equated to a reduction of 7.8 units for previous MDI patients and 4.8 units for previous tubed pump users. For an average pump patient, this is approximately a 10% reduction in total daily dose when moving from tubed pumps to the tubeless insulin management system.^3^ This reduction in insulin use may be attributed to both the form factor and features of the tubeless insulin management system which allow for more continuous insulin infusion and action. The tubeless nature of the device facilitates infusion site rotation as the Pod can be easily worn on multiple areas of the body compared to tubed pumps. The required Pod change at 72 hours eliminates prolonged infusion set wear beyond the 2 to 3 day recommended change, which often occurs with tubed pumps, and can result in both an increase in insulin use and hyperglycemia.^4^

Positive glycemic outcomes with tubeless insulin pump use have also been observed in a long-term retrospective analysis of the German/Austrian Diabetes Patienten Verlaufsdokumentation (DPV) registry.^5^ Danne and colleagues reported significantly lower HbA1c, 7.5% versus 7.7%, and TDD of insulin at 1 year in youth who switched to the tubeless insulin management system, compared to those who continued MDI use. In this large cohort of more than 2500 patients, TDD remained significantly lower with tubeless insulin pump use over 3 years compared to those who remained on MDI, with no difference in body mass index.^5^ The authors concluded that tubeless insulin pump therapy was an effective alternative to MDI for youth with type 1 diabetes.

#### Type 2 Diabetes

**Figure 5.**
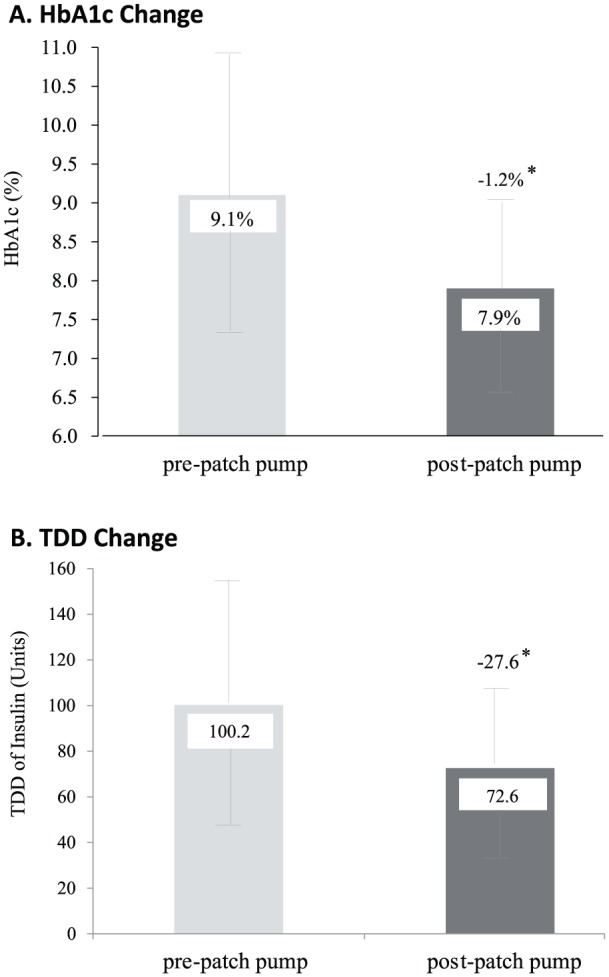
Change in HbA1c level and total daily dose (TDD) of insulin from baseline on MDI therapy at 3 months post–patch pump treatment initiation in patients with type 2 diabetes. (A) Decrease in HbA1c level (mean ± SD) of 1.2% ± 1.4% (–13 ± 15 mmol/mol) from baseline on MDI therapy (pre–patch pump) versus postpatch pump at 3 months. (B) Decrease in TDD (mean ± SD) of 27.6 ± 30.9 units of insulin from baseline on MDI therapy (prepatch pump) versus post– patch pump at 3 months (n = 80). *P **<** .001. Reprinted with permission from Layne et al.^8^

There is emerging evidence to support pump therapy for patients with type 2 diabetes who require insulin and those with high insulin daily doses who are not achieving adequate glycemic control.^6,7^ In a multicenter, retrospective study of 81 patients with type 2 diabetes commencing tubeless pump therapy, a reduction in HbA1c of 1.2% after 3 months was observed compared to previous treatment with MDI^8^ (Figure 5). In addition to the HbA1c reduction, an almost 30% reduction was seen in the average TDD of insulin from 100.2 to 72.6 units. These results have important potential clinical and economic impact for patients with type 2 diabetes and high insulin requirements and who seek improved glycemic control with CSII and prefer a discreet tubeless pump option.

### Do Tubeless Insulin Pump Users Report Positive Quality of Life?

In addition to improved glycemic outcomes, patients report improvement in quality of life (QOL) with use of the tubeless insulin management system compared to their previous treatment modality.^9^ In a recent survey study by Polonsky and colleagues of more than 1200 adults with type 1 diabetes using the tubeless insulin management system, there were QOL and clinical benefits associated with use of this system.^9^ The majority of patients reported positive changes in overall well-being (53.5%), perceived control over diabetes (72.5%), hypoglycemic safety (50.6%), and diabetes distress (69.6%). Improvement in glycemic control was also reported by the majority (64.2%) of respondents with 35.2% reporting a decrease in episodes of severe hypoglycemia. These data are particularly compelling as 44% of respondents had previously used a tubed pump.

### What Are the Long-Term Adherence Patterns With Tubeless Insulin Pump Use?

Ultimately, adherence is a surrogate marker for treatment satisfaction. In a recent, observational, retrospective study of 508 adults on pump therapy at a large tertiary center in the United Kingdom,^10^ Leelarathna and colleagues reported adherence rates with the tubeless insulin management system of 99% after 3 years for 120 patients. This retention rate was significantly higher than tubed pumps which ranged from 81% to 96% (*P* = .002). In this review, there was no difference in HbA1c reduction between tubed and tubeless pumps, with a reduction from 8.7% to 8.2% which led the authors to recommend that individual patient preference should be the key determinant in the choice of pump type.

### Can the Tubeless Insulin Pump Be Used in a Closed-Loop System?

A critical need in the diabetes community is safe and effective technologies that will reduce the physical and cognitive burden of diabetes care. Technological advances need to confer benefit as well as be discreet and reliable to reduce the burden of care. To this end, the Omnipod Horizon™ Automated Glucose Control hybrid closed-loop system is being developed. This system will leverage the technology developed for the Omnipod DASH System. The current tubeless Pod will contain a personalized model predictive control (MPC) based algorithm that will work in conjunction with the latest Dexcom CGM to allow automated insulin delivery. Initial feasibility studies of the MPC algorithm using an investigational device have yielded promising results.^11^ Buckingham et al. recently reported the results of a 36-hour inpatient hybrid closed-loop study of the personalized MPC algorithm in 58 patients aged 6 to 65 years. The algorithm performed well with approximately 70-73% of time spent in target range (70 to 180 mg/dL) and ⩽2% of time spent in hypoglycemia <70 mg/dL. Mean glucose ranged between 153 to 162 mg/dL across age groups. Additional trials are underway assessing the algorithm in free-living conditions across all age groups of patients with type 1 diabetes (NCT03064906, NCT03216460).^12^

## Conclusion

Insulin pump therapy is a widely used treatment method for diabetes for patients of all ages. There are several insulin pumps available, and patients should have the opportunity to choose the pump best suited for their needs to optimize treatment adherence, outcomes and patient engagement. The Omnipod Insulin Management System provides several differentiating features, including automated priming and cannula insertion, waterproof (IP28) Pod housing, and a tubeless wearable design. The Omnipod DASH System introduces several new features including a touchscreen PDM, Bluetooth wireless technology, a suite of mobile applications, and automatic integrated data management. This newly FDA-cleared insulin management system was carefully designed with extensive user input to be easy to use and maximize patient and caregiver engagement. Ultimately, better user engagement leads to enhanced treatment adherence and potentially improved clinical outcomes.
